# The impact of catheter ablation of atrial fibrillation on the left atrial volume and function: study using three-dimensional echocardiography

**DOI:** 10.1007/s10840-019-00696-8

**Published:** 2019-12-30

**Authors:** Jongmin Hwang, Hyoung-Seob Park, Seongwook Han, Seung-Woon Jun, Na-Young Kang, Jin-Hwa Jeon, Sang-Woong Choi, Cheol Hyun Lee, In-Cheol Kim, Yun-Kyeong Cho, Hyuck-Jun Yoon, Hyungseop Kim, Chang-Wook Nam, Seung-Ho Hur

**Affiliations:** Cardiovascular Center, Keimyung University Dongsan Hospital, 1035, Dalgubeol-daero, Daegu, Dalseo-gu, 42601 South Korea

**Keywords:** Atrial fibrillation, Catheter ablation, Left atrium, Atrial remodeling, Three-dimensional echocardiography

## Abstract

**Purpose:**

The exact correlation between the baseline left atrial (LA) volume (LAV) and atrial fibrillation (AF) radiofrequency catheter ablation (RFCA) outcomes and changes to the LA after AF RFCA has not yet been fully understood. We sought to evaluate the serial changes in the LAV and LA function after RFCA using 3D echocardiography.

**Methods:**

Consecutive patients who received RFCA of paroxysmal (PAF) or persistent AF (PeAF) at our center between January 2013 and March 2016 were included. Real-time 3D apical full-volume images were acquired, and a 3D volumetric assessment was performed using an automated three-beat averaging method. The LAV index (LAVI) was calculated and the LA ejection fraction (LAEF) was calculated as [LAVmax − LAVmin]/LAVmax.

**Results:**

Ninety-nine total patients were enrolled, and the mean age was 58.0 ± 8.2 years and 75 (74.7%) were male. There were 59 (59.6%) PAF patients and the remaining 40 (40.4%) had PeAF. AF recurred in 5 of 59 (8.5%) PAF and in 10 of 40 (25%) PeAF patients. The LAVImax increased on 1 day, decreased at 3 months, and then increased again at 1 year but was lower than that at baseline. The LAEF changes were similar to the volume changes but were more prominent in PeAF than PAF patients. The baseline 3D LAVImax was an independent predictor of AF recurrence after RFCA and the cut-off value was 44.13 ml/m^2^.

**Conclusion:**

In our study, even after 3 months of scar formation due to ablation, structural remodeling of the LA continued. The changes were more prominent in the non-recurrent, PeAF patients.

**Electronic supplementary material:**

The online version of this article (10.1007/s10840-019-00696-8) contains supplementary material, which is available to authorized users.

## Introduction

Atrial fibrillation (AF) is common and associated with an elevated risk for strokes and death [1, 2]. The pathogenesis of AF remains incompletely understood. Recent studies revealed that triggering events and maintenance substrate with clinical modulating factors such as obesity, tobacco, hypertension, and obstructive sleep apnea are comprising the complex mechanism of AF [3]. Especially, the initiation and maintenance of AF mainly depend on the left atrium (LA). Indeed, many studies have reported the positive association between the occurrence/recurrence of AF and an increased LA size/impaired LA function [4–6]. Therefore, an accurate evaluation of the LA is important to characterize individuals with or at risk of AF and to evaluate the strategies to treat or prevent AF. Three-dimensional (3D) echocardiography has shown a close correlation and minimal bias compared with cardiac magnetic resonance imaging for the measurement of the LA volume (LAV) and is superior to two-dimensional (2D) echocardiography because image foreshortening can be overcome [7, 8]. Radiofrequency catheter ablation (RFCA) of AF is now considered a cornerstone therapy for drug-refractory symptomatic AF. Previous studies have demonstrated a decrease in the LA dimensions and improved LA function after catheter ablation, but the majority of the studies has been based on 2D echocardiography [9–11]. In this study, we evaluated the serial changes in the LA volume and LA function using 3D echocardiography to determine how the LA in AF patients changes after catheter ablation. Using this data, we investigated the impact of catheter ablation of AF on the LAV and LA function.

## Methods

### Study population and protocol

We enrolled consecutive patients who received RFCA for drug-refractory paroxysmal or persistent AF at our hospital from January 2013 to March 2016. Paroxysmal AF (PAF) was defined as the occurrence of two or more episodes of AF during the previous year, all of which terminated spontaneously within1 week. Persistent AF (PeAF) was defined as continuous AF lasting more than 7 days. Because the purpose of our study was to elucidate the changes in the LAV after AF RFCA, an echocardiographic assessment on the same or previous day of the ablation, and then 1 day/3 months/1 year after the ablation (1 day, 3 months, 1 year), was planned. The exclusion criteria were as follows: prior AF ablation, prior cardiac surgery, significant valvular heart disease, severely decreased left ventricular (LV) ejection fraction (EF) (< 40%), hypertrophic cardiomyopathy, pulmonary disease, thyroid disease, and patients with severe procedural complications requiring surgery. Finally, 99 patients were enrolled and completed the follow-up. All patients signed their informed consent for the procedure, and the study was approved by the Institutional Review Board of Keimyung University Dongsan Hospital.

### Echocardiographic studies

A comprehensive echocardiographic examination (Siemens ACUSON SC2000, 4Z1c real-time volume imaging transducer [2.5 MHz]) was performed in all patients. The investigators were blinded to the study outcomes. The conventional standard echocardiographic parameters were obtained based on the American Society of Echocardiography (ASE) guidelines [12]. As described by the guidelines, the LA diameter was measured at end-systole from the parasternal long-axis view and a 2D volumetric assessment was performed using the biplane area-length method: the LA maximal volume (LAVmax) and LA minimal volume (LAVmin) were measured at the end of ventricular systole and diastole, respectively. A 3D volumetric assessment was performed using an automated three-beat averaging method. Real-time 3D apical full-volume images were acquired. All image data were analyzed using eSie analysis software, which is an offline, dedicated SC2000 workplace system (Siemens Medical Solution, Mountain View, CA, USA) (detailed method of acquiring 3D LAV are described in supplementary materials). The LA volume index (LAVI) was calculated as the LAV/body surface area, and the LA ejection fraction (LAEF) was calculated as [LAVmax − LAVmin]/LAVmax. Mainly, the 3D LAVI was used for the volumetric evaluation, and the LAEF was used for the functional evaluation.

### Ablation procedure

Multislice computed tomography (CT) scan was performed 1 day before or the day of the procedure to assess for any structural heart disease. Further, transesophageal echocardiography (TTE) was performed in all patients to rule out any LA/LA appendage thrombi. During the TTE, a baseline 2D examination was initially performed, and then a detailed analysis of the LAV was performed using the 2D and 3D examination methods as described above.

The RFCA procedures were performed under a fasting state with conscious sedation after withdrawal from anti-arrhythmic drugs for a period equal to five-times the half-lives of the drugs. Three multipolar catheters were inserted through the left femoral region and placed in the right atrium, coronary sinus, and His bundle. Two 8.5 Fr nonsteerable long sheaths (Swartz™ Braided Transseptal Guiding Introducer SL1, Abbott, St. Paul, MN) were placed in the right atrium through the right femoral region and inserted into the LA using one or two transseptal punctures. After the transseptal puncture, an activated clotting time of over 350 s was maintained with intermittent boluses of intravenous heparin. The anatomical structure was identified using LA and pulmonary vein (PV) angiography, and a 3D mapping system (CARTO® 3, Biosense Webster, Diamond Bar, CA, USA, or EnSite™ Velocity™, Abbott/St. Jude Medical, St. Paul, MN, USA) was used to map the anatomical structure of the LA and PVs. Then, the prescanned CT and 3D images were merged. Intracardiac electrograms were obtained using a Prucka CardioLab™ Electrophysiology system (General Electric Marquette, Inc., Milwaukee, WI, USA).

Initially, a point-by-point ablation was conducted at the junction between the LA and PVs to achieve a complete four pulmonary vein isolation (PVI) under guidance with a 20-pole circular mapping catheter (LASSO®, Biosense Webster, Inc., Diamond Bar, CA or Inquiry Optima™, Abbott/St. Jude Medical, St. Paul, MN, USA). Then additional lines and/or a non-PV trigger ablation were performed according to the physicians’ discretion. The radiofrequency energy was delivered using an irrigated ablation catheter (THERMOCOOL® SMARTTOUCH™ catheter, Biosense Webster, Inc., Diamond Bar, CA or M-Curve IBI Therapy CoolFlex™, Abbott/St. Jude Medical, St. Paul, MN, USA).

### Follow-up

Anti-arrhythmic agents were allowed during the first 3 months of the blanking period, and the maintenance of AADs after the blanking period was left to the physicians’ discretion. An ECG and 24-h Holter monitoring were obtained right after the procedure and at 3, 6, and 12 months after the ablation. ECG and Holter monitoring were also performed whenever the patient had symptoms suggesting an arrhythmia recurrence. Recurrence of atrial arrhythmias was defined as the detection of AF or an atrial tachycardia lasting more than 30 s on the ECG or 24-h Holter monitoring.

### Statistical analysis

Continuous variables are expressed as the mean value ± standard deviation. Categorical variables are expressed as numbers and percentages. Differences between the echocardiographic data for each patient at the different time points of the assessment were examined with two-tailed paired Student’s *t* tests. The difference in the serial changes of the LAVI and differences between groups were tested using a repeated measures analysis of variance (RM-ANOVA, Greenhouse and Geisser method was used for corrections based upon the estimates of sphericity). A receiver-operating characteristics (ROC) curve analysis was performed to evaluate the optimal cut-off value of the baseline LAVI for the prediction of a recurrence. All statistical analyses were performed using the MedCalc software package, version 18.11.3 (MedCalc Software, Mariakerke, Belgium). A *p* value < 0.05 was considered statistically significant.

## Results

### Clinical and procedural characteristics

The study population was composed of 99 consecutive drug-refractory AF patients. The mean age was 58.0 ± 8.2 years and 75 (74.7%) were male. There were 59 PAF patients (59.6%), and the remaining 40 (40.4%) had PeAF. The mean CHA_2_DS_2_-VASc score was 1.0 ± 1.1, mean LV EF, 65.0 ± 6.2%, and two patients had an LVEF below 50% (42 and 44%, respectively). Class I anti-arrhythmic drugs were used in 60 patients (60.6%) and class III anti-arrhythmic drugs were used in 17 patients (17.2%). A successful circumferential four PV isolation was performed in all patients and only a PV isolation was performed in 18 (18.2%, PAF 11, PeAF 7) patients. An additional line creation was performed as follows: cavotricuspid isthmus ablation in 81 (81.8%, PAF 48, PeAF 33), LA roof ablation in 13 (13.1%, PAF 4, PeAF 9), mitral isthmus ablation in 12 (12.1%, PAF 3, PeAF 9), SVC isolation in 4 (4.0%, PAF 4), and defragmentation of fractionated electrograms in 3 (3.0%, PAF 2, PeAF 1). During a 1-year follow-up period, AF or atrial tachycardia recurred in 5 of 59 (8.5%) PAF patients and 10 of 40 (25%) PeAF patients after the blanking period. And 54.5% of patients were using amiodarone (20,) or class Ic anti-arrhythmic drugs (38,) after the blanking period. The results of the baseline clinical characteristics are summarized in Table 1.

**Table 1:** Baseline characteristics

Characteristic	Total *N* = 99
Male	75 (74.7)
Age (year)	58.0 ± 8.2
Body mass index (kg/m^2^)	25.1 ± 2.5
Type of AF	
Paroxysmal	59 (59.6)
Persistent	40 (40.4)
CHA_2_DS_2_-VASc score	1.0 ± 1.1
Score 0–1	72 (72.7)
Score ≥ 2	27 (27.3)
Patients with LV EF < 50%	2 (2.0)
History of hypertension	28 (28.3)
History of diabetes	13 (13.1)
Serum creatinine	1.0 ± 0.9
Creatinine 1.2~1.5	7 (7.1)
On hemodialysis	2 (2.0)
AF recurrence	15
Paroxysmal	5 (8.5)
Persistent	10 (25)
Duration of anticoagulation before TEE (days)^†^	37 (34, 50)
Echocardiographic findings	
LV EF (%)	65.0 ± 6.2
LA volume_max_ (ml)*	84.4 ± 26.8
LA volume index_max_ (ml/m^2^)*	47.2 ± 16.2
Anti-arrhythmics after blanking period	
Amiodarone	20 (20.2)
Class Ic	38 (38.4)

Values are presented as *n* (%) or mean ± SD. *AF* atrial fibrillation, *LV* left ventricle, *EF* ejection fraction, *TIA* transient ischemic attack, *LA* left atrium, *SEC* spontaneous echo contrast, *TEE* transesophageal echocardiography

†Duration of anticoagulation is presented as median (interquartile range)

*Three-dimensionally measured

### Echocardiographic results

#### Change in the LA volume after AF ablation

**Fig. 1:**
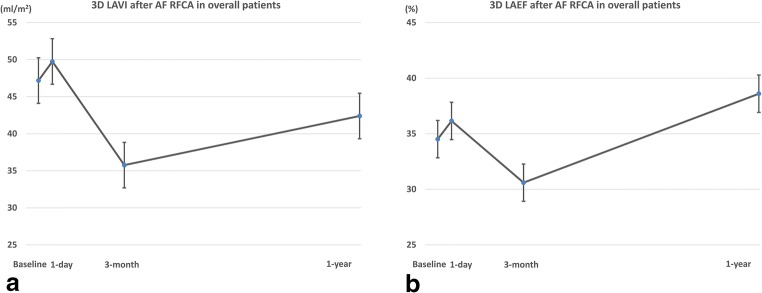
Change in left atrial volume index (LAVImax) and ejection fraction (EF) of overall patients after atrial fibrillation ablation. **a** Change of LAVImax. The LAVImax increased by 1 day, decreased at 3 months, and then increased again at 1 year. **b** Change of LAEF. The changing pattern of in the LAEF was similar to that of the volume change

In our measurements, the volume of the LA exhibited a dynamic change during the year of the post-ablation period. Compared with the baseline, the LAVImax increased by 1 day, decreased at 3 months, and then increased again at 1 year, but the 1 year LAVImax was lower than that at baseline (3D LAVImax; baseline: 47.2 ± 16.2, 1 day: 49.8 ± 14.7, 3 months: 35.8 ± 10.8, 1 year: 42.4 ± 13.2, Fig. 1a). This changing pattern regarding the time and difference between the measurements were statistically significant (within subjects *P* < 0.0001, pairwise comparisons between measurements were all statistically significant. Data are shown in Table 2).

**Table 2:** Pairwise comparisons of LA volume indexes between each follow-up points using three-dimensional echocardiography

Factors		Mean difference	Std. error	*P* value	95% CI
Baseline	1 day	− 5.205	1.476	0.0040	− 9.188 to − 1.223
	3 months	20.653	1.824	< 0.0001	15.732 to 25.575
	1 year	9.756	1.870	< 0.0001	4.713 to 14.800
1 day	Baseline	5.205	1.476	0.0040	1.223 to 9.188
	3 months	25.859	1.805	< 0.0001	20.988 to 30.729
	1 year	14.962	1.872	< 0.0001	9.912 to 20.011
3 months	Baseline	− 20.653	1.824	< 0.0001	− 25.575 to − 15.732
	1 day	− 25.859	1.805	< 0.0001	− 30.729 to − 20.988
	1 year	− 10.897	1.503	< 0.0001	− 14.953 to − 6.842
1 year	Baseline	− 9.756	1.870	< 0.0001	− 14.800 to − 4.713
	1 day	− 14.962	1.872	< 0.0001	− 20.011 to − 9.912
	3 months	10.897	1.503	< 0.0001	6.842 to 14.953

#### Change in the LAEF after AF ablation

For a functional assessment, the LAEF was calculated and compared. When calculated using 3D volume measurements, the changing pattern in the LAEF in the total patients also exhibited dynamic changes and the pattern was similar to that of the volume change: increased by 1 day, decreased at 3 months, and increased again at 1 year (LA EF; baseline: 34.5 ± 11.4, 1 day: 36.2 ± 9.7, 3 months: 30.6 ± 7.1, 1 year: 38.6 ± 8.8, the changing pattern regarding the time was statistically significant, within subjects *P* < 0.001, Fig. 1b).

#### Changes in the LA volume and LAEF according to the AF pattern

##### PAF vs. PeAF

**Fig. 2:**
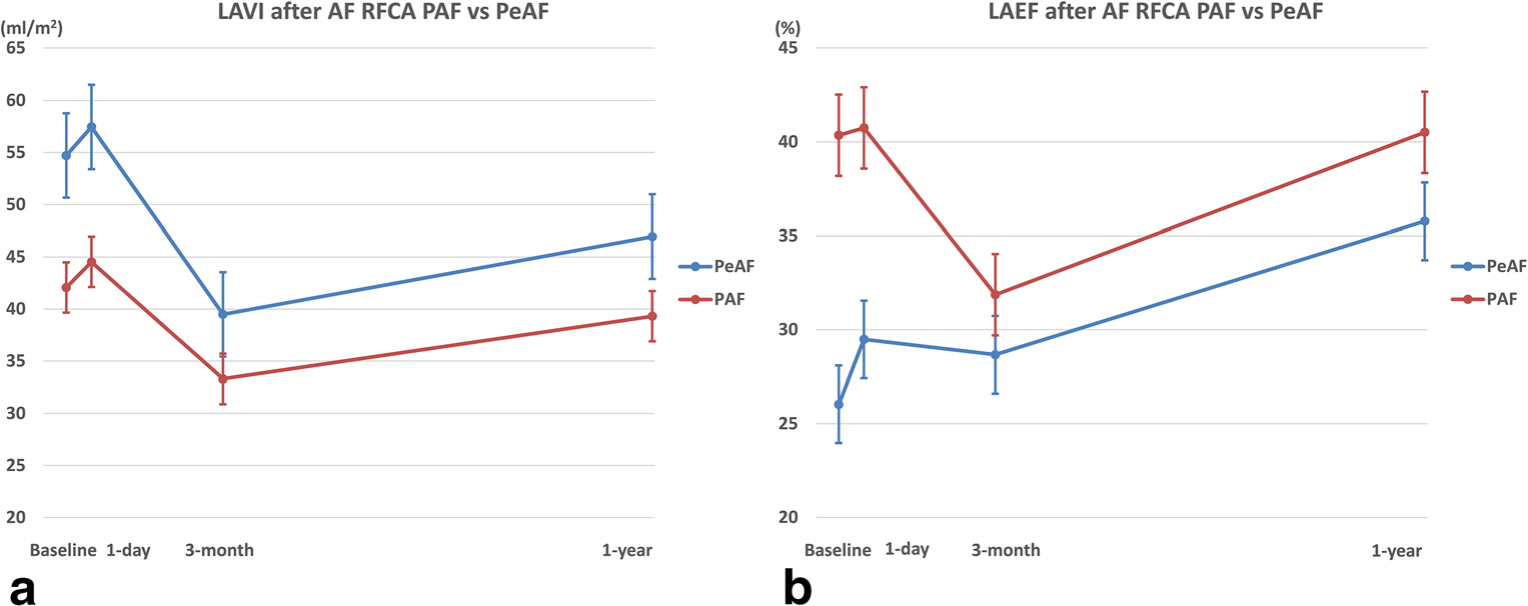
Comparisons of change in left atrial volume index (LAVImax) and ejection fraction (EF) after atrial fibrillation (AF) ablation between paroxysmal (PAF) and persistent AF (PeAF) patients. **a** Change of LAVImax. The changing patterns in the LAVImax in both the PAF and PeAF patients were similar to those in the overall patients, but the mean value was lower in the PAF patients than PeAF patients. **b** Change of LAEF. The LAEF in the PeAF patients slightly increased by 1 day, slightly decreased at 3 months, and then increased much more than that at baseline at 1 year

The changing patterns in the LAVImax in both the PAF and PeAF patients were similar to those in the overall patients, but the mean value was lower in the PAF patients than PeAF patients. However, the LAEF had a different pattern. In the PAF patients, the LAEF slightly increased by 1 day, decreased at 3 months, and recovered to the baseline level at 1 year. On the other hand, the LAEF in the PeAF patients slightly increased by 1 day, slightly decreased at 3 months, and then increased much more than that at baseline at 1 year. Especially, the PeAF patients exhibited a nearly continuous increase in the LAEF during the follow-up period. The mean value of the LAEF was higher in the PAF patients than PeAF patients (*P* < 0.001, Fig. 2). An RM-ANOVA analysis revealed a change in the LAVI and LAEF regarding the group, time, and group-time interaction, and it was statistically significant, which meant that the serial change in the LAVI and LAEF was significant, and the changing pattern between the groups also significantly differed (between group: LAVI *P* < 0.001, LAEF *P* < 0.001, within group: LAVI *P* < 0.001, LAEF *P* < 0.001, group-time interaction: LAVI *P* = 0.003, LAEF *P* < 0.001).

##### Non-recurrent vs. recurrent group

**Fig. 3:**
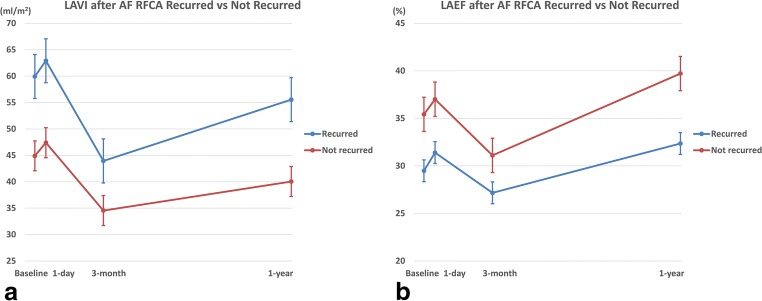
Comparisons of change in left atrial volume index (LAVImax) and ejection fraction (EF) after atrial fibrillation ablation between recurrent and non-recurrent patients. **a** Change of LAVImax. The LAVImax decreased by 3 months and increased by 1 year regardless of a recurrence. **b** Change of LAEF. The changing patterns in the LAEF were also not related to the recurrence

In the volume measurements, the LAVImax decreased by 3 months and increased by 1 year regardless of a recurrence. Further, also the changing patterns in the LA EF were not related to the recurrence. However, the absolute values differed (the LAVImax was higher and LAEF lower in the recurrent group than non-recurrent group [Fig. 3]). An RM-ANOVA analysis revealed changes in the LAVI and LAEF regarding the group and time was statistically significant, but the group-time interaction was not significant, that is the changing patterns in two groups were similar according to the time (between group LAVI *P* < 0.001, LAEF *P* = 0.005, within group LAVI *P* < 0.001, LAEF *P* = 0.001, group-time interaction LAVI *P* = 0.236, LAEF *P* = 0.419).

### Baseline factors predicting an AF recurrence

**Fig. 4:**
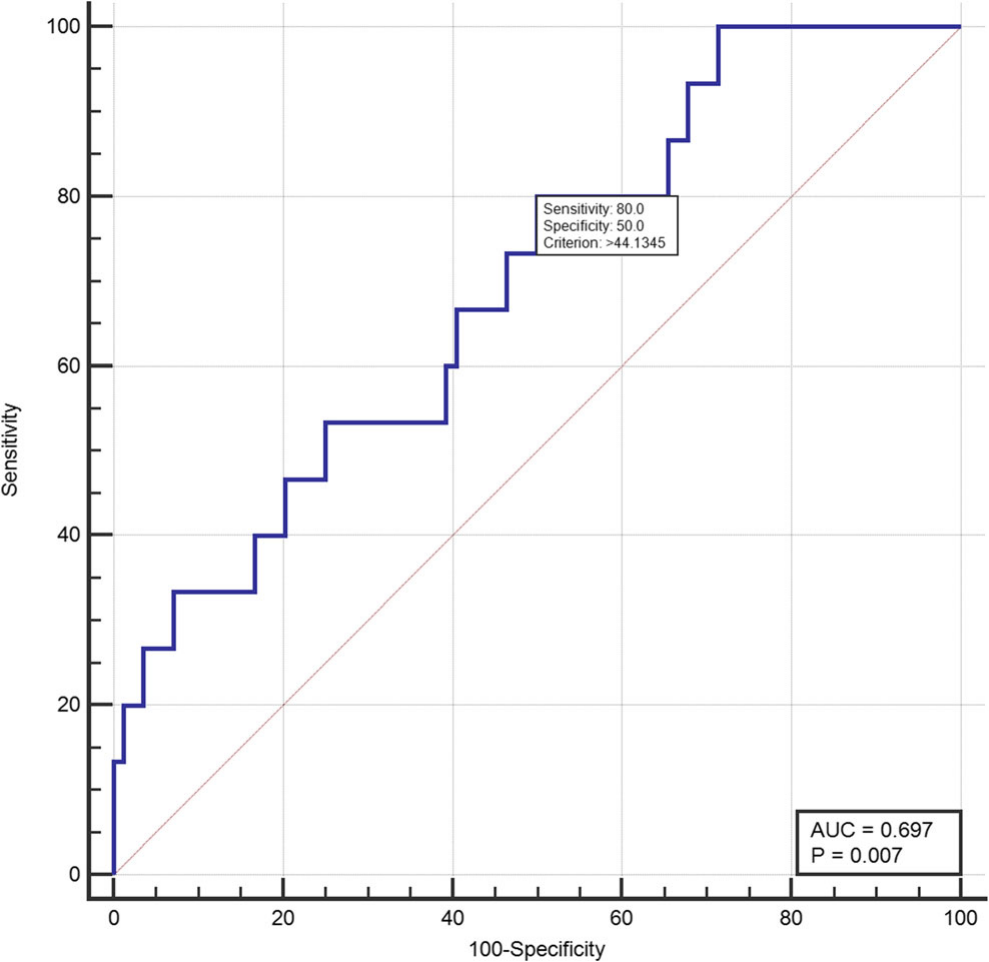
Receiver-operating characteristics (ROC) curve analysis of LAVImax for recurrence after atrial fibrillation ablation. A cut-off value of 44.13 ml/m2 predicted an AF recurrence with an 80.0% sensitivity and 50.0% specificity (area under the curve = 0.697, *P* = 0.007).

In the univariate analysis, persistent AF and 3D LAVI were predictors of an AF recurrence. The age, gender, body mass index, hypertension, and diabetes did not reach a statistical significance. However, in the multivariate analysis, the baseline 3D LAVImax was confirmed as the only independent predictor of an AF recurrence (*P* = 0.049, OR = 1.04, 95% CI 1.00–1.08, Table 3). The ROC curve analysis revealed that the discriminating ability of the 3D LAVImax for an AF recurrence was fair and a cut-off value of 44.13 ml/m^2^ predicted an AF recurrence with an 80.0% sensitivity and 50.0% specificity (area under the curve = 0.697, 95% CI 0.596–0.785, Youden index *J* = 0.3; *P* = 0.007, Fig.4).

**Table 3:** Multivariate analysis for the baseline characteristics which predict recurrence of AF after RFCA

	Univariate analysis		Multivariate analysis^†^	
	OR (95% CI)	*P* value	OR (95% CI)	*P* value
Male	0.44 (0.14–1.39)	0.161		
Age	0.99 (0.93–1.06)	0.829		
Body mass index	0.85 (0.68–1.06)	0.147		
Persistent AF	3.6 (1.13–11.51)	0.031*		
History of hypertension	1.88 (0.60–5.89)	0.279		
History of diabetes	0.43 (0.05–3.57)	0.433		
Baseline 3D LAVImax	1.06 (1.02–1.09)	0.004*	1.05 (1.02–1.09)	0.004*

*AF* atrial fibrillation, RFCA radiofrequency catheter ablation, OR odds ratio, CI confidence interval, *3D LAVImax* three-dimensional (3D) echocardiographic measurement of left atrial volume index

^†^Stepwise method for variable selection

*Statistical significance

## Discussion

### Main findings

**Fig. 5:**
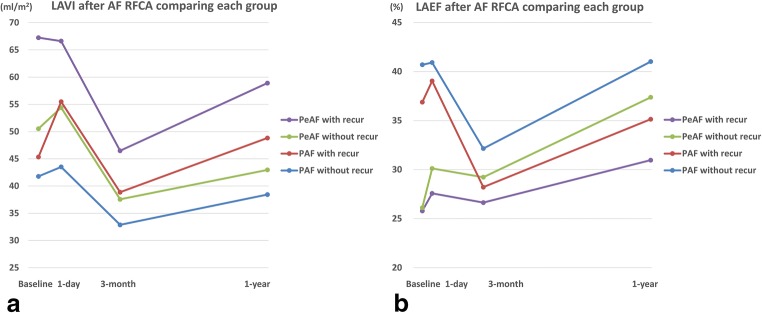
Comparisons of change in left atrial volume index (LAVImax) and ejection fraction (EF) after atrial fibrillation (AF) ablation between subgroups. **a** Change of LAVImax. The changing patterns in each subgroup were similar to that in the overall patients regardless of the type (paroxysmal AF vs. persistent AF) or recurrence. **b** Change of LAEF. In the PeAF patients, the LAEF continuously increased after RFCA and the patients without recurrence had a more dramatic change. On the other hand, in the PAF patients, the LAEF was markedly decreased at 3 months and returned to the baseline level at 1 year

In the present study, we used the 3D echocardiographic method to assess the LAV and LA function after RFCA of AF. The major findings of this study were as follows: (1) The LAVI in the AF patients exhibited dynamic changes after RFCA. Compared with the baseline, the LAVI slightly increased by 1 day, decreased at 3 months, and then increased again at 1 year, but was lower than that at baseline. (2) For the LAVI, the changing patterns in each subgroup were similar to that in the overall patients regardless of the type (PAV vs. PeAF) or recurrence. However, the mean value was higher in the PeAF/recurrent patients than in the PAF/non-recurrent patients (Fig. 5a). (3) The overall pattern of the LAEF changes was similar to that of the LAVI, but a subgroup analysis showed a different pattern. In the PeAF patients, the LAEF continuously increased after RFCA and the patients without recurrence had a more dramatic change. On the other hand, in the PAF patients, the LAEF was markedly decreased at 3 months and returned to the baseline level at 1 year. The mean values were higher in the non-recurrent group than in the recurrent group (Fig. 5b). (4) In the multivariate analysis, the baseline 3D LAVImax was an independent predictor of an AF recurrence after RFCA and a cut-off value of 44.13 ml/m^2^ yielding the best discrimination.

### LA volume and AF, AF RFCA

A large body of evidence indicates that a large LA size/volume is associated with new-onset AF and a poor outcome of AF RFCA [6, 13, 14]. However, the exact correlation between the baseline LA size/volume and AF RFCA outcome and the serial changes in the LA after AF RFCA has not yet been fully established. Our study confirmed that the average LA volume during the follow-up period was lower in PAF patients and non-recurrent patients than in those patients without, as in the previous studies (Fig. 4) [6].

One of the unique features of our study was the observation that the LA volume was constantly changing during the 1-year follow-up period. Although there was a difference in the degree of the changes according to the subgroup, the LA volume increased immediately after the procedure but decreased at 3 months and then increased again at 1 year. In particular, an increase in the LA volume on the day after the procedure was observed, which was not revealed in the previous studies. The transient atrial contractile dysfunction following cardioversion, also known as atrial “stunning,” may mainly contribute to this phenomenon but this transient increase in LA volume was also observed in PAF patients. Hence, a tissue edema/inflammation, effect of autonomic nerve modulation, increase in the circulating volume due to the use of irrigated ablation catheters, or another unknown mechanism may also contribute to this. In addition, the maximal LA volume/size changes were achieved at 3 months, and then LA remodeling proceeded after that in our study. This phenomenon also has not been well described in the previous studies, and after 3 months of scar formation, structural remodeling of the LA continued regardless of the subgroup, indicating that AF is a progressive disease. Therefore, it is necessary to monitor the patient continuously after the 3-month blanking period from the RFCA, and a careful judgment is needed, especially for anticoagulation therapy and anti-arrhythmic agent use.

### Impact of AF RFCA on the LA size and function

In almost all studies, a decrease in the LA volume after AF RFCA has been confirmed, but it is still under investigation as to whether this leads to an increase in the LA function and whether the reduction in the volume is the result of reverse remodeling or scar formation [15]. Recently, several reports have been published showing that patients who underwent additional substrate modification and a longer duration of radiofrequency energy deliveries had a greater decline in the LA volume and greater increase in the LA pressure as compared with those patients with a pulmonary vein isolation only [16, 17].

Because we aimed to find a simple and practical method for the risk stratification of patients with AF, we did not perform a detailed functional study of the LA. Instead, we simply calculated the LAEF using the maximal/minimal LA volume, and it has been used as a surrogate marker for the LA function in several previous studies [11, 18, 19]. As with the volume, the mean LAEF exhibited a dynamic change during the follow-up period and was higher in the PAF/non-recurrent patients than in the PeAF/recurrent patients. Notably, the changes in the LAEF were more prominent in the PeAF patients than PAF patients, especially in the non-recurrent PeAF patients. Although the LAEF calculated in our study may not have been the most accurate assessment of the LA function, at least, maintaining sinus rhythm with catheter ablation seems to have had a greater impact on the PeAF patients than PAF patients. Compared with the other studies, our study enrolled a relatively large number of PeAF patients.

### Factors predicting recurrence of AF after RFCA

Now, there is no consensus on which modality is the best method to evaluate the LA in AF patients. In this regard, our study was based on the hypothesis that assessing the LA volume using 3D echocardiography can be a simple and accurate method compared with the other methods. Previous small studies showed promising results for real-time 3D echocardiography [20, 21], and our study showed that the baseline LAVI measured by 3D echocardiography was an independent predictor of a recurrence of AF after RFCA. In our analysis, the 2D and 3D had a very high correlation in the volume measurements (Supplement Table 1), but only the 3D LAVI was an independent predictor of an AF recurrence. Therefore, we recommend using the 3D echocardiographic method for an LA evaluation in AF patients. Also, given the limitations, it is meaningful that our research has revealed that the recurrence of AF can be predicted with only echocardiographic volume measurements without a complicated evaluation.

In our study, anti-arrhythmic drugs (AAD) were used after the blanking period according to the physician’s discretion. Interestingly, the post-blanking period AAD use was significantly associated with recurrence of AF after RFCA (Univariate odds ratio 6.82, *p* value 0.015). This is contrary to the current concept and the results of recent randomized study [22]. Indeed, analysis of baseline characteristics of patients on AAD revealed that they were more prone to recurrence of AF; proportion of persistent AF patients were higher in AAD group (no AAD vs AAD: 28.9% (13/45) vs 50% (27/54), chi-square *p* value 0.034), and value of baseline 3D LA volume index was higher in AAD group (no AAD vs AAD: 43.1 ± 13.5 vs 50.6 ± 17.5, independent sample *t* test p value 0.02). This may be due to the biased decision of physicians that prescribing AAD to those with a high risk of recur, which demonstrates the limitations of non-randomized trial.

### Limitations

The non-randomized enrollment with a relatively small number of patients was a major limitation of our study. Baseline characteristics between patients with PAF and PeAF were different, which in turn may impact the study results derived in the current study. However, it was difficult to separate the study population into PAF and PeAF cohorts for meaningful statistical analysis with the small population size. The PAF patients underwent measurements during sinus rhythm and the PeAF patients during the AF state, which would also be a major limitation, but rather it reflected the more real-world practice. The timing of the echocardiographic exam was also limited, and we could have observed more dynamic LA volume changes at 6 months or 9 months after the procedure, and over 1 year after the procedure.

## Conclusion

After catheter ablation of AF, the LA showed continuous structural and functional remodeling. This was more prominent in PeAF patients, and it is speculated that the main effect of AF RFCA is trigger control in PAF patients and structural remodeling in PeAF patients. The LAVI obtained by 3D echocardiography was more helpful than 2D measurement or simple volume measurement in AF RFCA outcome prediction.

## Electronic supplementary material

ESM 1 (JPG 101 kb)

ESM 2 (JPG 83 kb)

ESM 3 (JPG 80 kb)

ESM 4 (DOCX 22 kb)

ESM 5 (AVI 1756 kb)

ESM 6 (MP4 8197 kb)
