# Supplementary figures and images for: The impact of catheter ablation of atrial fibrillation on the left atrial volume and function: study using three-dimensional echocardiography
Source: J Interv Card Electrophysiol. 2019 Dec 30;57(1):87–95. doi: 10.1007/s10840-019-00696-8 (PMC7036070; doi:10.1007/s10840-019-00696-8)

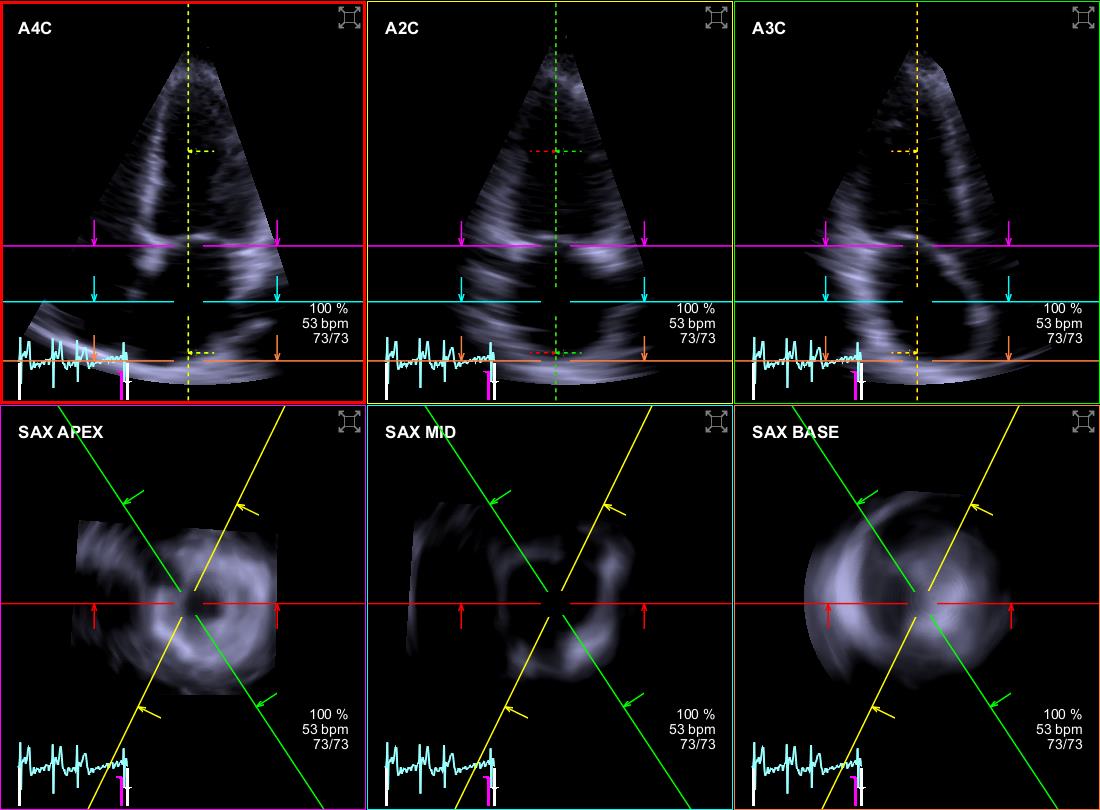

Supplement: Supplementary file 1 — (JPG 101 kb) [file 10840_2019_696_MOESM1_ESM.jpg]

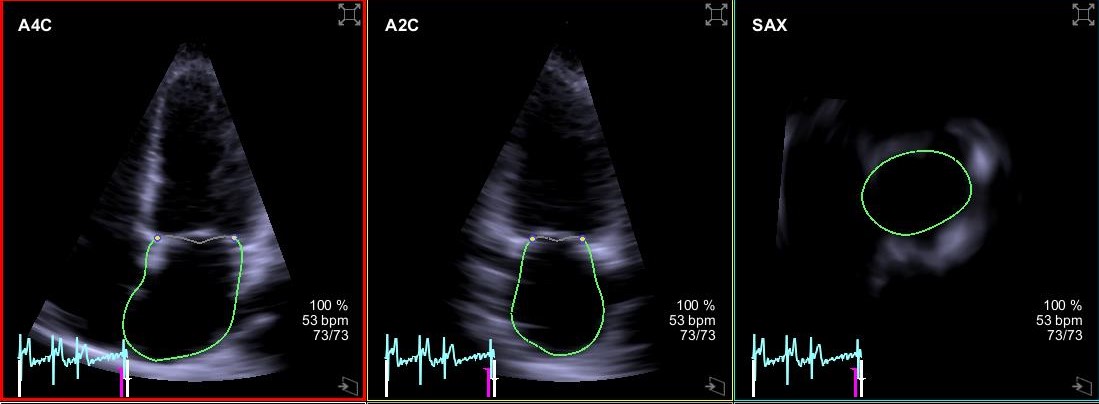

Supplement: Supplementary file 2 — (JPG 83 kb) [file 10840_2019_696_MOESM2_ESM.jpg]

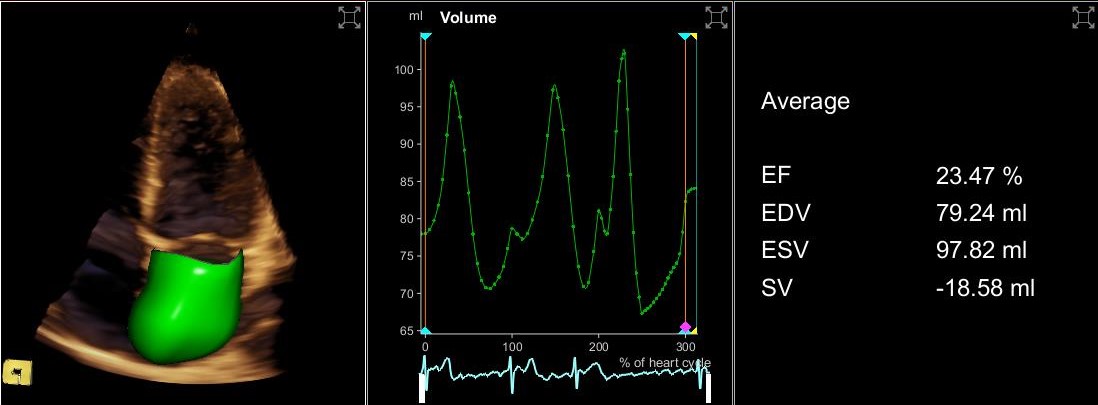

Supplement: Supplementary file 3 — (JPG 80 kb) [file 10840_2019_696_MOESM3_ESM.jpg]
