# Supplementary material for: The impact of catheter ablation of atrial fibrillation on the left atrial volume and function: study using three-dimensional echocardiography
Source: J Interv Card Electrophysiol. 2019 Dec 30;57(1):87–95. doi: 10.1007/s10840-019-00696-8 (PMC7036070; doi:10.1007/s10840-019-00696-8)
Supplement: Supplementary file 4 — (DOCX 22 kb) [file 10840_2019_696_MOESM4_ESM.docx]

**Table 1. Correlation table between baseline measurements**

|  | | Baseline_2D_LAD | Baseline_2D_LAV | Baseline_2D_LAVI | Baseline_3D_LAV | Baseline_3D_LAVI |
| --- | --- | --- | --- | --- | --- | --- |
| Baseline_2D_LAD | Correlation coefficient Significance Level P n |  | 0.719 <0.0001 99 | 0.659 <0.0001 99 | 0.690 <0.0001 98 | 0.609 <0.0001 99 |
| Baseline_2D_LAV | Correlation coefficient Significance Level P n | 0.719 <0.0001 99 |  | 0.953 <0.0001 99 | 0.944 <0.0001 98 | 0.892 <0.0001 99 |
| Baseline_2D_LAVI | Correlation coefficient Significance Level P n | 0.659 <0.0001 99 | 0.953 <0.0001 99 |  | 0.893 <0.0001 98 | 0.924 <0.0001 99 |
| Baseline_3D_LAV | Correlation coefficient Significance Level P n | 0.690 <0.0001 98 | 0.944 <0.0001 98 | 0.893 <0.0001 98 |  | 0.959 <0.0001 98 |
| Baseline_3D_LAVI | Correlation coefficient Significance Level P n | 0.609 <0.0001 99 | 0.892 <0.0001 99 | 0.924 <0.0001 99 | 0.959 <0.0001 98 |  |

**Methods - 3D LA volume acquirement[1]**

The measurements were performed via averaging three consecutive beats, aiming to obtain maximal LA volume at end-systole. The procedure performed with the Siemens ACUSON SC2000, 4Z1c real-time volume imaging transducer (2.5 MHz). All image data were analyzed using eSie analysis software which is an offline, dedicated SC2000 workplace system (Siemens Medical Solution, Mountain View, CA, USA).

The process of obtaining 3D LA volume performed sequentially as following;

1. Real-time 3D apical full-volume images were acquired (Supplement Video 1)

2. Left atrium was focused in apical full-volume image (Supplement Figure 1).

3. The software created a geometric model of LA through manual designation of the mitral annulus and superior dome point in the frames corresponding to end-diastolic and end-systolic time; subsequent border detection was performed based on an automated algorithm that detected the endocardial wall interface (Supplement Figure 2). (This automated identification was based on pattern recognition learning from large annotated data repositories. This technology also allows for automated delineation (auto-contouring) of the endocardium of the LA throughout the entire cardiac cycle. (Supplement video 2))

4. In the following step, the contours were manually corrected if necessary. The pulmonary vein orifices and LA appendage were not included in the contour.

5. Then automatically calculated maximal LA volume/EF was reported (Supplement Figure 3).ㄴ

**Reference**

1. Heo R, Hong GR, Kim YJ, Mancina J, Cho IJ, Shim CY et al. Automated quantification of left atrial size using three-beat averaging real-time three dimensional Echocardiography in patients with atrial fibrillation. Cardiovasc Ultrasound. 2015;13:38. doi:10.1186/s12947-015-0032-5.
